# Disseminated histoplasmosis diagnosed on a blood smear in a Nigerian patient with non-Hodgkin’s lymphoma

**DOI:** 10.1093/omcr/omae116

**Published:** 2024-10-10

**Authors:** Ofonime O Benjamin, Omolabake E Riman, Anthony Offiong, Walter O Egbara, Asukwo Onukak, Aje N Ogar, Bassey E Ekeng

**Affiliations:** Department of Haematology and Blood Transfusion, University of Calabar Teaching Hospital, Calabar, 540242, Nigeria; Department of Haematology and Blood Transfusion, University of Calabar Teaching Hospital, Calabar, 540242, Nigeria; Department of Medical Microbiology and Parasitology, University of Calabar Teaching Hospital, Calabar, 540242, Nigeria; Department of Internal Medicine, University of Calabar Teaching Hospital, Calabar, 540242, Nigeria; Department of Internal Medicine, University of Uyo, Uyo, 520001, Nigeria; Department of Haematology and Blood Transfusion, University of Calabar Teaching Hospital, Calabar, 540242, Nigeria; Department of Medical Microbiology and Parasitology, University of Calabar Teaching Hospital, Calabar, 540242, Nigeria

**Keywords:** blood smear, disseminated histoplasmosis, HIV/AIDS, lymphoma, immunocompromised

## Abstract

Disseminated histoplasmosis is rarely reported in patients living with cancers in Nigeria. We report a 40-year-old woman who presented with left neck swelling and abdominal pain of two weeks duration. Clinical examination and radiological findings showed pallor, epigastric tenderness, generalized lymphadenopathy and hepatosplenomegaly. An initial diagnosis of sepsis and micronutrient deficiency was made following findings of macrocytosis, hypersegmented neutrophils and toxic granulations on blood smear. Intervention with antibiotics did not improve symptoms rather her clinical presentation worsened with the onset of fever, dizziness, easy fatiguability and generalized weakness. Histology of lymph node biopsy reported a diffuse large B-cell lymphoma. A repeat examination of the blood smear revealed budding yeast cells morphologically similar to *Histoplasma capsulatum*. This case emphasizes the need for a high index of suspicion of histoplasmosis in this at-risk population and the usefulness of a blood smear in diagnosing histoplasmosis.

## Introduction

Histoplasmosis is not an uncommon clinical entity in Nigeria [1]. About 231 cases were reported between 1968 and 2022 [2]. However, there are no documented cases in patients living with cancers in Nigeria [2, 3]. This case report aims to drive awareness of histoplasmosis in patients living with hematological malignancies in Nigeria. In addition, it affirms the utility of a blood smear in diagnosing histoplasmosis, especially in a setting like ours where fungal diagnostics are not routinely available [4].

## Case report

A 40-year-old woman presented with complaints of left neck swelling of a month duration and abdominal pain of two weeks duration. Abdominal pain was in the epigastric region, aggravated with meals and relieved by analgesics. The patient had a history of peptic ulcer disease and underlying HIV infection and was receiving highly active antiretroviral therapy, including Tenofovir, Dolutegravir, and Lamivudine once daily.

Essential findings on general examination were pallor, generalized lymphadenopathy, afebrile with a temperature of 36.9°C and tenderness in the epigastric region. The spleen was palpable 12 cm below the left costal margin. She had a blood pressure of 120/80 mmHg but with accompanying tachypnea, tachycardia and haemic murmurs. An urgent hematocrit done was 20%. A clinical diagnosis of acute exacerbation of peptic ulcer disease with imminent anaemic heart failure in an immunocompromised patient was made. She was transfused with 2 units of pack red cells and was thereafter placed on tablets omeprazole, 20 mg b.d and suspension Gascol for 10 days. Abdominal pain resolved within 48 hours following the commencement of the medications.

Results of investigations done two days after were a Full blood count which showed a white blood cell count of 3.4 × 10^3^/μl, neutrophil −0.77 × 10^3^/μl, lymphocytes −0.93 × 10^3^/μl, red blood cells −2.25 × 10^6^/μl, haemoglobin −6.5 g/dl, platelets −292 × 10^3^/μl, a hematocrit of 20.5% and an erythrocyte sedimentation rate of 165 mm/h. Significant findings on peripheral blood film were macrocytosis and hypersegmented neutrophils with toxic granulations. HIV viral load was 727 copies/ml with a CD4+ cell count of 675 cells/μl. An abdominal ultrasound scan showed hepatosplenomegaly. Liver function test showed a total bilirubin of 11.3 μmol/l, conjugated bilirubin −4.2 μmol/l, aspartate transaminase −87 μmol/l, and alanine transaminase of 19 -μmol/l. Gene Xpert was negative for *Mycobacterium tuberculosis*. Chest X-ray and serum electrolytes, urea and creatinine were not done due to financial constraints. The patient was being managed on an outpatient basis while awaiting the result of the cervical lymph node biopsy.

**Figure 1:**
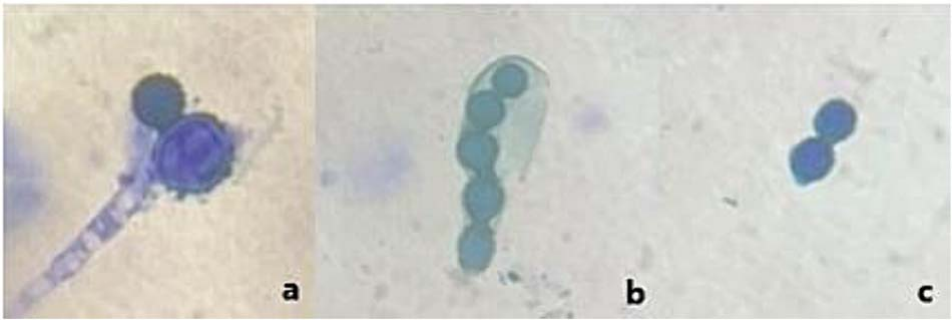
(**a**) Tuberculated macroconidia of *Histoplasma* borne on hyphae (**b**) Tuberculated macroconidia of *Histoplasma* transforming into yeast cells (**c**) Oval yeast cells of *Histoplasma*, budding on a narrow base.

Two weeks later she presented with a gaping wound at the biopsy site with purulent exudates and was admitted. She was then placed on intravenous ceftriaxone, gentamycin and metronidazole empirically. Four days into admission, she developed a high-grade continuous fever with temperature measurements ranging from 38.3°C to 40.3°C. Giemsa-stained blood smear microscopy was negative for malaria parasite. Wound swab and blood cultures were also negative. Histology of lymph node biopsy reported a diffuse large B-cell lymphoma. However, a repeat examination of the patient’s blood smear by a clinical microbiologist revealed tuberculated budding yeast-like structures morphologically like *Histoplasma capsulatum*, fig. 1. Amphotericin B was not accessible; hence patient was placed on 200 mg itraconazole, thrice a day while making efforts to find amphotericin B. Unfortunately, the patient declined further treatment and left against medical advice due to her inability to financially sustain her treatment.

## Discussion

We highlight perhaps the first reported case of disseminated histoplasmosis in a Nigerian patient with underlying malignancy. Although not a rare disease, case series and observational studies showed cases of histoplasmosis are significantly being misdiagnosed as tuberculosis and cancers [2, 5, 6]. Other reasons accounting for missed cases include a low index of suspicion for histoplasmosis amongst clinicians, lack of diagnostics, and the paucity of research on histoplasmosis in Nigeria [2, 4–8]. This brings to the fore the need to keep driving awareness of histoplasmosis in the Nigerian setting which has a significant proportion of its population at risk of fungal diseases including individuals living with HIV and or cancers.

Regarding the risk factors, there were no findings in the history of presenting complaints suggesting *Histoplasma* infection. She was not a farmer, no known exposure to excreta of bats or other activities that may warrant severe exposure to *Histoplasma* conidia. Her compromised immune system secondary to the lymphoma and HIV infection must have exposed her to histoplasmosis although the CD4+ cell count was not remarkably low.

The diagnosis of histoplasmosis can be done via serum or urine *Histoplasma* antigen assay which is highly sensitive for patients with disseminated disease [9]. Unfortunately, the kits are unavailable for routine use in our setting due to cost considerations. Bone marrow culture could not be done because following biopsy the incision site wasn’t healing despite receiving antibiotics probably because of the low white blood cell counts. Thus, bone marrow aspirate for culture was declined. Besides the diagnostic tools for *Histoplasma* antibody detection not being available, it has a low sensitivity in the diagnosis of disseminated histoplasmosis in patients with underlying HIV infection and so was not a preferred diagnostic tool in this scenario [9]. The diagnosis was made on a Giemsa blood smear which showed budding yeast cells morphologically similar to *Histoplasma capsulatum.* Its utility has been shown in several case reports and reviews [10]. One large-scale review identified 58 cases of disseminated histoplasmosis diagnosed on blood smear of which 53 (91.4%) were confirmed by classical diagnostic methods; (62.3% (33/53) by culture, 56.6% (30/53) by histopathology, 32.1% (17/53) by serology, 13.2% (7/53) by molecular detection and 3.8% (2/53) by cytology) [10]. We conclude that where the options for proven diagnosis are not available, a blood smear would be helpful and should be encouraged in a resource-limited setting like ours. However, this requires a trained microscopist as the diagnosis may be missed due to a lack of expertise. As seen in this index case, the diagnosis was made on a repeat blood smear by a clinical microbiologist.
